# Medical Science and Materialism

**Published:** 1854-03

**Authors:** 


*Medical Science and Materialism.* A Discourse delivered before the Faculty and Students of the Buffalo Medical College, on Sabbath the 12th Feb., 1854. By John C. Lord, Pastor of the Central Presbyterian Church. Published by request.

Among the best of the good things which it becomes our duty to notice this month, is this sermon of Dr. Lord's. It seems to us, and we know that other minds think with us, that this sermon contains one of the best arguments against materialism that we have ever perused. While it attacks materialism itself, it defends the profession from the unjust charge of a tendency to that heresy. It draws a beautiful contrast between the Christian medical philosopher and the unthinking atheist. The names of Good, of Haller, of Boerhaave, of Darwin, and other Christian physicians, are brought prominently forth as evidence of the elevating tendencies of medical science, and in shining contrast to the dark infidelity which has so often marked the student of the merely psychological sciences.

We like anything that is bold and manly — freely outspoken in that manner called in the vernacular "flat-footed." Such is one prominent merit in Dr. Lord's sermon. Aside from the clear logic of its argumentation, the beauty, and occasional sublimity of its rhetoric, it has the higher merit of being most unmistakable in its teachings and tendencies. There is no non-committalism, no softening down the matter for phrenological materialists, or homœpathic sisters. He asserts strongly the claim of medicine to a dignified rank as one of the learned professions; he strenuously objects to that throwing aside of foregone experience, and trusting in one-idea-ism, which so marks the age.

We are betrayed into this panegyric because it was the first time in our life when we have had the pleasure of hearing a distinguished divine denouncing, to a full house, the crying sin of quackery, openly advocating the claims of legitimate medicine, and casting the shafts of clerical argument at patent doctors, and patent medicines. From reading the following, we may almost hope for the restoration of the ancient comity of feeling between physic and divinity, which has been made so rare by the attempts of an emasculated clergy to add the supervision of the cure of bodies to that of souls:

"I need not dwell upon the antiquity of the medical profession. The most ancient Scriptures abound with references to the art of the physician. Some of the most eminent names that have come down to us from antiquity are of men belonging to your profession. The dignity of the calling to which you have devoted yourselves is seen both in its antiquity and necessity; for man, falling by transgression under the law of disease and death, has ever been compelled to resort to remedies and to seek the aid of those who have acquired skill and experience in the art of healing. The various casualties to which the human frame is exposed, require the office of the surgeon, whose success depends upon his intimate acquaintance with the human frame; the diseases which are his sad inheritance require the care, the skill, and the learning of those who have devoted themselves to the acquisition of the knowledge necessary to the relief of their fellow-men.

"The dignity of the medical profession is seen in the fact that it is one of the learned professions. It requires the knowledge of various sciences, it involves great responsibility, and demands both skill and judgment for its proper exercise. It is highly desirable that every medical student should have a thorough education in all departments of learning; but where this is impossible, it is the more necessary that there should be a thorough training in all that relates to medical science. The profession of medicine loses all its dignity, and, I may add, all its value, when it falls into the hands of empirics. Charlatanism and quackery abound in our age, with all its vaunted intelligence, and are nowhere more conspicuous than in the host of pretenders in the art of healing, who make a boast of their ignorance, and ground their claim to the confidence of the public on the fact that they do not practice after the usual mode; that they make no use of the experience of the past or of the lights of science. They have new medicines, new theories, and a host of marvelous cures, as their stock in trade, and their success can only be accounted for on the principle which has led men, in various ages and places, to use amulets and charms to cure diseases, and which induces the

American savage to admit that every pretender, who clothes himself with a little mystery, and makes conjuration, and talks in a way not to be understood, and boasts of a wonderful cure when he does not happen to kill—is a great 'medicine.' The real credulity of the age in which we live may be seen by the constant and successful appeals made to it by patent doctors and the inventors of patent medicines. Only by maintaining a high standard of education, by a conscientious discharge of their important duties, and by sternly refusing all connection with the empiricisms which surround them, can the faculty of medicine maintain the true dignity of their profession. 'Wisdom,' is said in the Scriptures, 'to be justified of her children;' and knowledge, experience and skill must, in the end, triumph over pretense and charlatanry. Let no student who seeks to enter upon the practice of medicine, for the sake of any temporary advantage, strike hands with men that he knows to be unlearned, or with systems that he knows to be unsound. Let him wait for success in the paths of honor and probity; for such success will be enduring, and accompanied by a good conscience toward God and men."

The class of the college have done well in publishing this sermon, and we shall hope for an extended circulation for it. S. B. H.

